# The World Health Organization COVID-19 surveillance database

**DOI:** 10.1186/s12939-022-01767-5

**Published:** 2022-11-23

**Authors:** Maya Allan, Maja Lièvre, Henry Laurenson-Schafer, Stéphane de Barros, Yuka Jinnai, Sophie Andrews, Thomas Stricker, Jesus Perez Formigo, Craig Schultz, Anne Perrocheau, Julia Fitzner

**Affiliations:** WHO-HQ WHE COVID-19 IMST, Geneva, Switzerland

**Keywords:** SARS-CoV-2, COVID-19, Population-based surveillance, Pandemic, Repository, Public health, Database, Disaggregation, Equity, Health care workers

## Abstract

In January 2020, SARS-CoV-2 virus was identified as a cause of an outbreak in China. The disease quickly spread worldwide, and the World Health Organization (WHO) declared the pandemic in March 2020.

From the first notifications of spread of the disease, the WHO’s Emergency Programme implemented a global COVID-19 surveillance system in coordination with all WHO regional offices. The system aimed to monitor the spread of the epidemic over countries and across population groups, severity of the disease and risk factors, and the impact of control measures. COVID-19 surveillance data reported to WHO is a combination of case-based data and weekly aggregated data, focusing on a minimum global dataset for cases and deaths including disaggregation by age, sex, occupation as a Health Care Worker, as well as number of cases tested, and number of cases newly admitted for hospitalization. These disaggregations aim to monitor inequities in COVID-19 distribution and risk factors among population groups.

SARS-CoV-2 epidemic waves continue to sweep the world; as of March 2022, over 445 million cases and 6 million deaths have been reported worldwide. Of these, over 327 million cases (74%) have been reported in the WHO surveillance database, of which 255 million cases (57%) are disaggregated by age and sex. A public dashboard has been made available to visualize trends, age distributions, sex ratios, along with testing and hospitalization rates. It includes a feature to download the underlying dataset.

This paper will describe the data flows, database, and frontend public dashboard, as well as the challenges experienced in data acquisition, curation and compilation and the lessons learnt in overcoming these. Two years after the pandemic was declared, COVID-19 continues to spread and is still considered a Public Health Emergency of International Concern (PHEIC). While WHO regional and country offices have demonstrated tremendous adaptability and commitment to process COVID-19 surveillance data, lessons learnt from this major event will serve to enhance capacity and preparedness at every level, as well as institutional empowerment that may lead to greater sharing of public health evidence during a PHEIC, with a focus on equity.

## Background

### History of COVID-19 and surveillance data collection

Global infectious disease surveillance is part of WHO’s mandate since the creation of the institution in 1948. The International Health Regulations (IHR) aim to standardize the list of a few epidemic prone diseases under immediate notification, as well as unexpected events of international concern to prevent international spread. The latest update of IHR in 2005 [1] included a list of diseases for immediate notification, definition of public health event of international concern requiring immediate notification, as well as a frame for an efficient communication between Member States and WHO. The list of epidemic prone diseases requiring immediate notification is adapted to national context. International surveillance existed for tuberculosis, smallpox, cholera, typhoid, flu, and the HIV pandemic during accelerated the development of national surveillance systems and data sharing. For acute respiratory disease with pandemic potential, the Global Influenza Surveillance and Response System [2], (GISRS) is operational since 1952, with a network of institutions in 124 countries, aiming to provide surveillance data on influenza and respiratory syndromes (Influenza Like Illness, Severe Acute Respiratory Infection).

In January 2020, SARS-CoV-2 was identified as the virus involved in an outbreak in the province of Hubei, China, as mentioned in the Disease Outbreak News [3]. The disease quickly spread worldwide, and the World Health Organization’s (WHO) Emergency Committee declared a Public Health Event of International Concern (PHEIC) on 30 January 2020 [4]. On 11 March 2020 [5], WHO declared COVID-19 as a pandemic. From the first notification of spread of the disease outside China, WHO’s Emergency Programme implemented a global COVID-19 surveillance system based on the IHR [6].

WHO’s response to previous PHEICs has provided valuable lessons and capacity building in preparedness for future emergencies. For example, in 2004, WHO guidelines for the Global Surveillance of SARS [7] recommended a minimum global dataset for surveillance, including case-based data and age and sex disaggregated data. Following this, the response to the 2009 influenza A (H1N1) pandemic emphasized the need for timely surveillance data to enable evidence-based decisions in public health [8]. From end of April 2009 to August 2010, WHO IHR contact points in WHO regional offices and IHR National Focal Points collected paper and/or electronic case-based clinical and epidemiological data from laboratory-confirmed cases. WHO headquarters (WHO-HQ) received these data operationalized as different variables and in different formats. Over 18,000 individual case reports of laboratory-confirmed influenza cases were reported to WHO from 84 of its 193 Member States of over 6 million cases allegedly reported worldwide. In comparison, considering the enormous number of cases over a much smaller period, surveillance of the COVID-19 pandemic proved to be an unprecedented challenge.

COVID-19 global surveillance aims to monitor the extension of the pandemic across countries, the severity of the disease and risk factors, and the impact of control measures. Data collection tools and standardized surveillance methods were documented in the interim guidance Public Health Surveillance for SARS-CoV-2 [9].

Standardized surveillance methods are designed to provided standard definitions, variable formats, and data collection tools to be able to merge surveillance datasets from different sources and maintain comparability and data compatibility. This is particularly of importance during a global pandemic such as COVID-19, with large case numbers, and cases and deaths reported from all over the world.

Updates of the interim guidance Public Health Surveillance for SARS-CoV-2 were released as evidence unfolded (nine such updates have been published up between January 2020 and February 2022). These updates involved notably case definition and laboratory confirmation methods, definition of reinfection, list of signs and symptoms included in case definition, inclusion of variables on vaccine status of cases.

Two years after the beginning of the COVID-19 pandemic, many aspects of epidemiological surveillance remain challenging despite many achievements and lessons learnt. This paper documents the construction of the WHO-HQ COVID-19 surveillance database by describing its data collection, storage, and dissemination mechanisms, followed by a discussion of the use of these data in facilitating analysis of inequalities. Finally, we discuss challenges and lessons learnt as well as future directions and proposed developments for the database.

### Construction

**Fig. 1:**
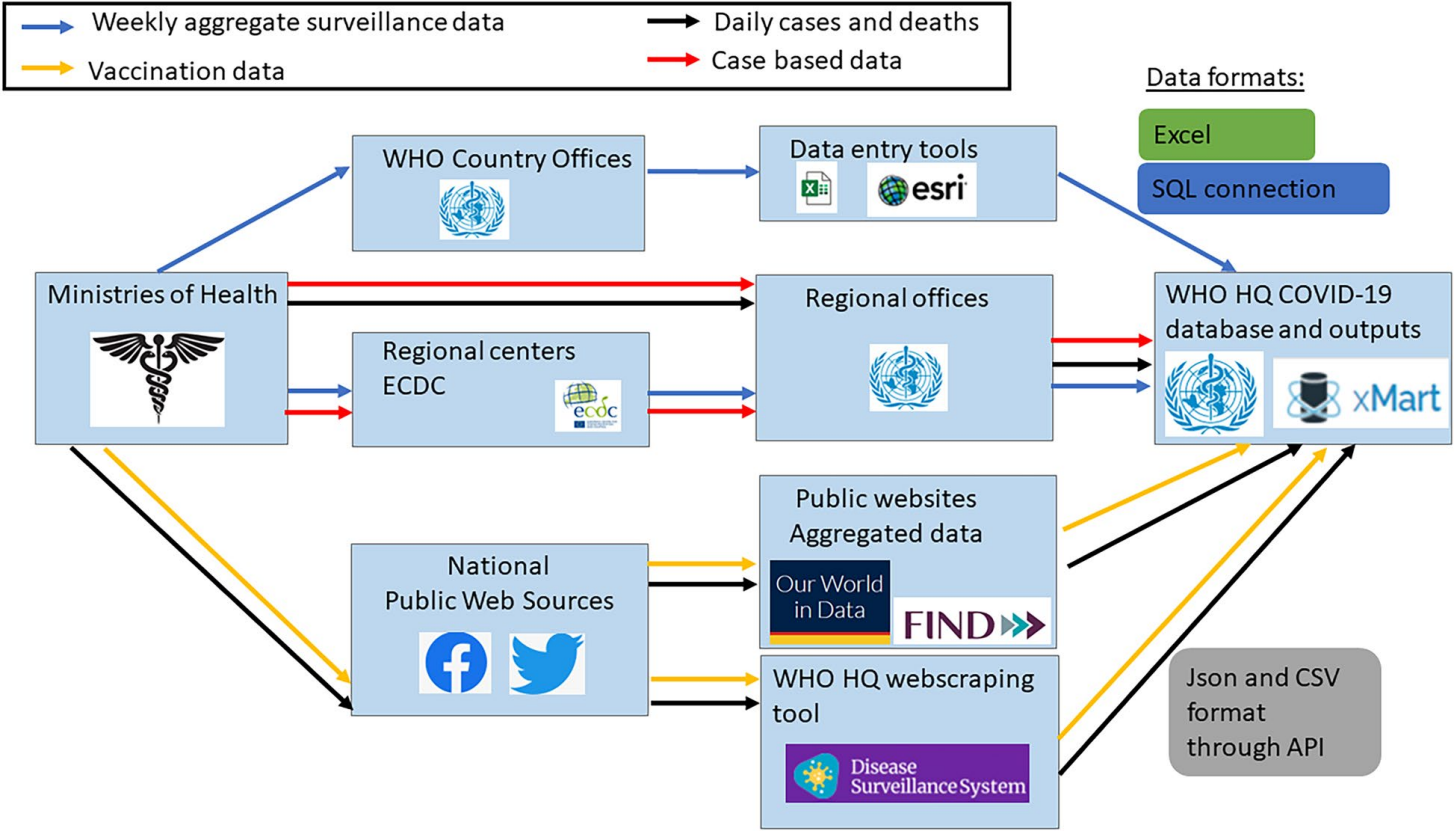
WHO-HQ COVID-19 surveillance data base: data flow. *Source: WHO-HQ COVID-19 IMST Epi Pillar Data management team*

Data flow of COVID-19 surveillance data from Ministries of Health to WHO-HQ is complex, and combines multiple sources, tools, and stakeholders (Fig. 1). Despite widespread advocacy on standardized surveillance tools, Ministries of Health often rely on existing systems, that do not always include recommended standards. This leads to multiple datasets, using different data formats, rendering major challenges in interoperability and dataset consolidation. This article focuses on the database maintained and coordinated at WHO-HQ, hereafter referred to as “WHO-HQ COVID-19 surveillance database”. The data acquisition, curation and analysis will be detailed in the paragraphs below.

### Data acquisition

In January 2020, WHO developed a COVID-19 case definition for surveillance and asked all countries to report cases through the IHR national focal points. WHO also started to collect and compile daily cases and death counts globally from web based public sources (public dashboards and social media) [10]. In the Interim guidance public health surveillance for SARS-CoV-2 published in August 2020, it was recommended that ministries of health share the case definition for COVID-19 case confirmation and deaths. However, there was little compliance from the Ministries of Health, and active follow-up on public websites to review case definitions.

The initial surveillance strategy aimed at collecting daily case-based data through a case report form [11], with standardized variables. The case-based data collection system was set up using existing regional surveillance networks. In several countries the case-based data was collected through the existing influenza surveillance system and in others via the routine disease surveillance system.

The influenza surveillance system was operational in 149 countries in 2019, where a network of a subset of sentinel health facilities reports Influenza like Illnesses and Severe Acute Respiratory Infection. In the beginning of the COVID-19 pandemic, emergency surveillance standards from IHR recommend reporting comprehensively all detected cases, from all health facilities, including those not included in the sentinel network. The influenza surveillance data management systems for case-based reporting, Flu ID, and Tessy, managed by ECDC in Europe, were adapted to report comprehensively all COVID-19 cases. In addition to this the virological reporting system (FluNet) was adapted to include SARS-Cov-2 detections by week in specimens received at the laboratory. In many countries, routine disease surveillance systems were adapted include COVID-19 in the list of mandatory diseases report, and the frequency of reporting was increased from weekly to daily reporting.

The objectives of the surveillance were to monitor areas and population affected by COVID-19 and to understand the epidemiological characteristics of the illness (incubation period, secondary attack rate, serial interval, case fatality rate) including age, sex, co-morbidities, settings of transmission, and nature of contacts to identify vulnerable groups at highest risk of exposure or severe disease. Variables recommended by WHO for COVID-19 surveillance evolved in accordance with latest scientific evidence on the pathogen (variants), response (vaccination), and impact on mortality, morbidity, and healthcare capacity. Geographical distribution of cases was reported at national and regional level. Localization of cases reported in case-based data was described at locality level, but challenges with data formatting, poor completeness and data curation rendered poor added value to the geographical information reported to WHO-HQ.

As the disease spread and numbers of cases increased, the burden on surveillance systems impacted the capacity to provide detailed case-based data, and in March 2020, recommendations for surveillance data reporting shifted to weekly aggregate reporting of a minimum global dataset comprising age and sex disaggregation of cases and deaths, newly hospitalized cases, and occupation (to capture cases among Health Care Workers (HCW). The set of variables for weekly aggregate reporting was further completed and amended in August 2020 [12] (see Table 1).

**Table 1:** Variables and sources contributing to WHO-HQ COVID-19 surveillance database

Surveillance variables	Weekly aggregated format		Other data sources				
	Weekly aggregated Surveillance data V1 March 2020	Weekly aggregated Surveillance data V2 August 2020	Case report form	Daily case counts	Our World in Data	FIND	Vaccination dataset
Confirmed and probable cases	**X**	**X**	**X**	**X**	**X**		
Confirmed and probable deaths	**X**	**X**	**X**	**X**	**X**		
Sex at birth of cases and deaths	**X**^**a**^	**X**	**X**				
Age of cases and deaths^b^	**X**	**X**	**X**				
Newly hospitalized	**X**	**X**	**X**				
Discharged (confirmed and probable)	**X**	**X**					
Cases in Health Care Workers	**XXX**	**X**	**X**				
Deaths of Health Care Workers	**X**	**XXX**	**X**				
Sex of Health Care Worker			**X**				
Number of people tested with any diagnostic method		**X**			**X**	**X**	
Number of people tested using only the PCR assay		**X**					
Total doses administered							**X**
Number of persons vaccinated with at least one dose							**X**
Number of persons fully vaccinated							**X**
Vaccines in use (names and brands)							**X**
Date of first vaccination rollout (by country)							**X**

^a^Sex disaggregation reported as the proportion of cases and deaths, rather than numbers

^b^Age disaggregation for cases and deaths also evolved over time. In the latest version, the following disaggregations for confirmed and probable cases and deaths are available: 0–4; 5–14; 15–24; 24–34; 35–44; 45–54; 55–64; 65–74; 75–84; 85+; unknown

**Fig. 2:**
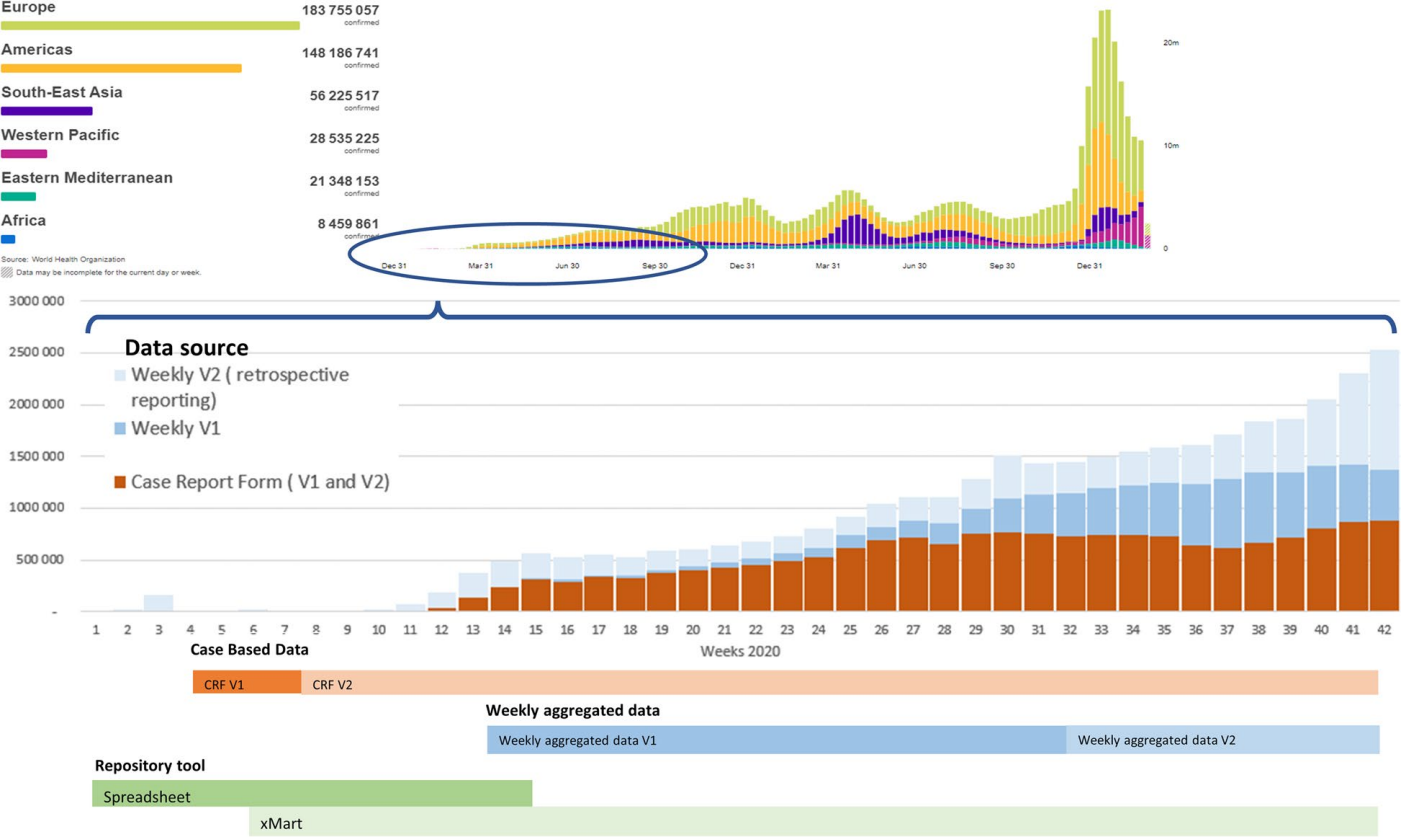
Milestones in early set up of WHO-HQ COVID-19 surveillance

Recommended variables and disaggregation of COVID-19 surveillance data allow for detection and reporting on changes in the disease in the affection between men and women, and in different subgroups such as infants, children, elderly, and other high-risk groups defined by occupational such as HCWs [13], social or behavioural factors, migrants, young adults and others. Inequality monitoring in the most vulnerable groups is a priority focus. Milestones of the adaptation of surveillance recommendations are displayed in Fig. 2.

While some countries and regions shifted to aggregate reporting as recommended by WHO, others continued to report case report forms throughout the pandemic. Some regions reported case-based data to the global database while others shared aggregated case-based data according to the recommended minimum dataset. Ad hoc data acquisition, aggregation and combination using various formats is also performed as some countries have not yet stabilised the data collection and aggregation process. Publicly reported data processed through global aggregators like “FIND” [14] and “Our World in Data” [15] were also loaded and used to complement the datasets reported by countries.

Internal analyses were performed to attempt to predict increases in transmission and anticipate operations and preventive measures in countries where the health systems would risk reaching capacity. These were kept internal given political sensitivity. Modelling techniques were tested by researchers to account for under reporting, but given wide variety of situations, factors, and highly dynamic situations, validation delays were not adapted to real time surveillance data received by WHO.

As vaccinations became available, WHO started to roll out data collection based on public websites in January 2021. A weekly system was implemented to aggregate the number of total administered doses, number of persons with at least one dose, and number of persons fully vaccinated. Additionally, data on types of vaccines used and date of vaccination rollout were collected. A monthly system of collecting more detailed data and including disaggregation by age and sex was launched based on the existing Joint Reporting Form used by WHO and UNICEF to collect immunization data from countries [16].

Given this diversity, data processing at WHO-HQ adapted accordingly, allowing for data collection through different means including form-based reporting, uploads of spreadsheets to the data platform, application programming interface (API) or direct server connections.

### Data curation and storage

At the beginning of the epidemic, the case-based data was stored anonymously on public data storage tools. With rising case counts and to use a more sustainable solution it was decided to use the WHO xMart system already used in the WHO Global Influenza Programme for the storage of case-based data. As more datasets were added when new reporting recommendations were issued, WHO xMART became the main repository for all COVID-19 surveillance data. Datasets were loaded regardless of the original format and transformed to fit the proposed data model. Regional office focal points were trained on how to upload data to WHO xMart and cut-off time was communicated to ensure timeliness of reported data. The WHO xMART system allowed to continuously adapt the global surveillance system to rapidly changing environment during the pandemic: to add and withdraw variables, to merge data from different formats, to aggregate individual and aggregated surveillance data, to change age-group distribution several times. Adaptability of data acquisition and storage capacity is essential while setting up an ad hoc surveillance system during a pandemic of a new emerging disease and xMART was a major asset.

Data from Ministries of Health were in general curated at regional offices before they were uploaded to the global database, except for some instances when the data were reported directly from the country to the global database. Automated data corrections were implemented in the global database including mostly curation of dates in case report forms and checks across fields to correct or complete missing information based on information from other fields, e.g. the calculation of age based on date of birth; and checking for dates to be in chronological order, e,g, date of death not being before date of onset. Furthermore, data quality control dashboards were made available to WHO data managers at WHO-HQ and regional offices. The latter provide overviews on data completeness and highlight data inconsistencies.

The surveillance data from the different formats and datasets were merged into one single dataset using an algorithm to enable maximal data availability for each recommended variable, without double counting. Data were combined by country, week, age and sex as available in the following priority order:

1. Data collected through weekly aggregated reporting (V2) [12]
2. Data collected through reporting systems recommended at earlier stages of the epidemic (weekly aggregated reporting (V1), case report forms [11])
3. Aggregated data from WHO regions
4. Aggregated testing data from FIND [14] (testing data only for countries, areas or territories in the WHO African Region)
5. Aggregated data from Our World in Data [15]

Age bands have changed in recommendations for aggregate reporting over time (age groups 15–24 and 24–64 in weekly aggregated V1 and age group 20–29 in Weekly Aggregate V2). To combine the data reported in both different age bands for analysis, national age-stratified population data was used to distribute the cases and deaths accordingly for each country following the approach used by the Max Planck Institute for their COVerAGE-DB [17].

The number of cases as reported through case report forms was aggregated to weeks using the date of report. Data from global daily case counts were used as reference to check for completeness of surveillance data received [10]. Timely sharing of data was prioritized over extensive data quality control. This has resulted in historical data changing when corrections have been applied post hoc, i.e., after the data has first been shared.

### Data analysis and dissemination

At the beginning of the epidemic, surveillance data was not disseminated publicly, but rather analyzed and internally disseminated at WHO. It was also used for risk assessments and detailed analyses, some of which were made publicly available in the WHO Weekly Epi Update [3].

In March 2021, a public dashboard displaying the main trends and features in variables in the WHO COVID-19 surveillance database was released [18]. The dashboard comprised eight views reporting cases and deaths disaggregated by age and sex (as well as for HCWs specifically), hospitalization, and testing. A download feature for the database, allowing the public to access the whole dataset, was added in April 2021. Data in the public dashboard are updated once every day for aggregated data sources and once every week for case-based data. Vaccination data, also stored on the WHO xMart, is displayed on a separate dashboard on the WHO website [19].

**Fig. 3:**
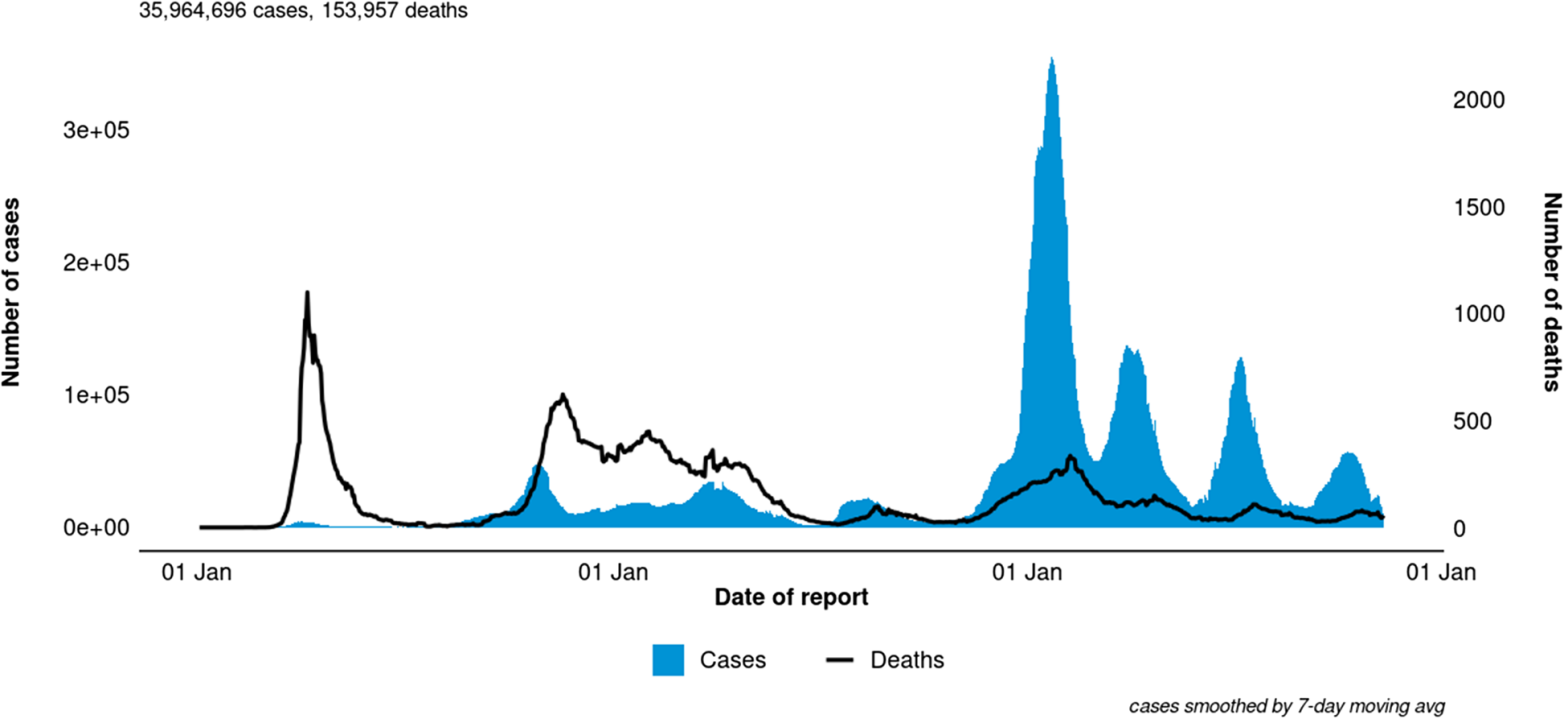
Example of the epidemiological curve of COVID-19 cases and deaths in a select country from the WHO COVID-19 Dashboard

Within the public dashboard, epidemiological curves displaying COVID-19 cases and deaths across time allow longitudinal analysis of trends, enhancing identification of departures from trends and decoupling of trends that can indicate shifts in transmission and severity dynamics, and impact of Non Pharmaceutical Interventions (NPI) and Public Health and Social Measures (PHSM) (Fig. 3).

**Fig. 4:**
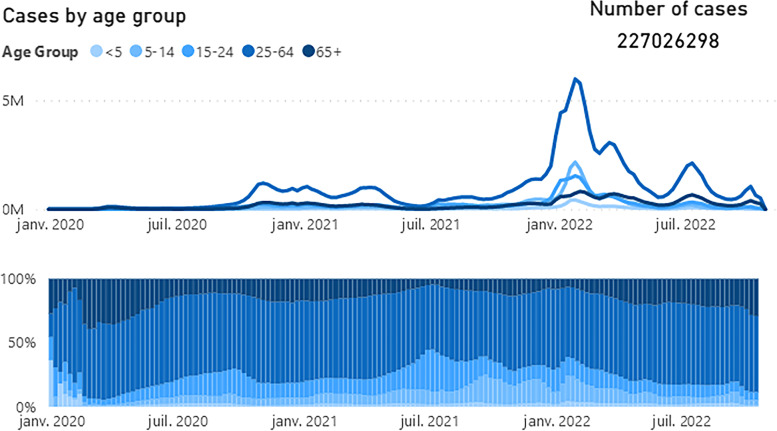
Example of age disaggregation of cases and deaths visualization from the WHO COVID-19 Dashboard

Age disaggregation of cases and deaths is displayed both in absolute numbers and in relative proportion (Fig. 4). The trend analysis of age disaggregation allows the identification of shifts in infection, hospitalisation, deaths and vaccination rollout and coverage across age groups, and both Non Pharmaceutical Interventions (NPI) and Public Health and Social Measures (PSHM).

**Fig. 5:**
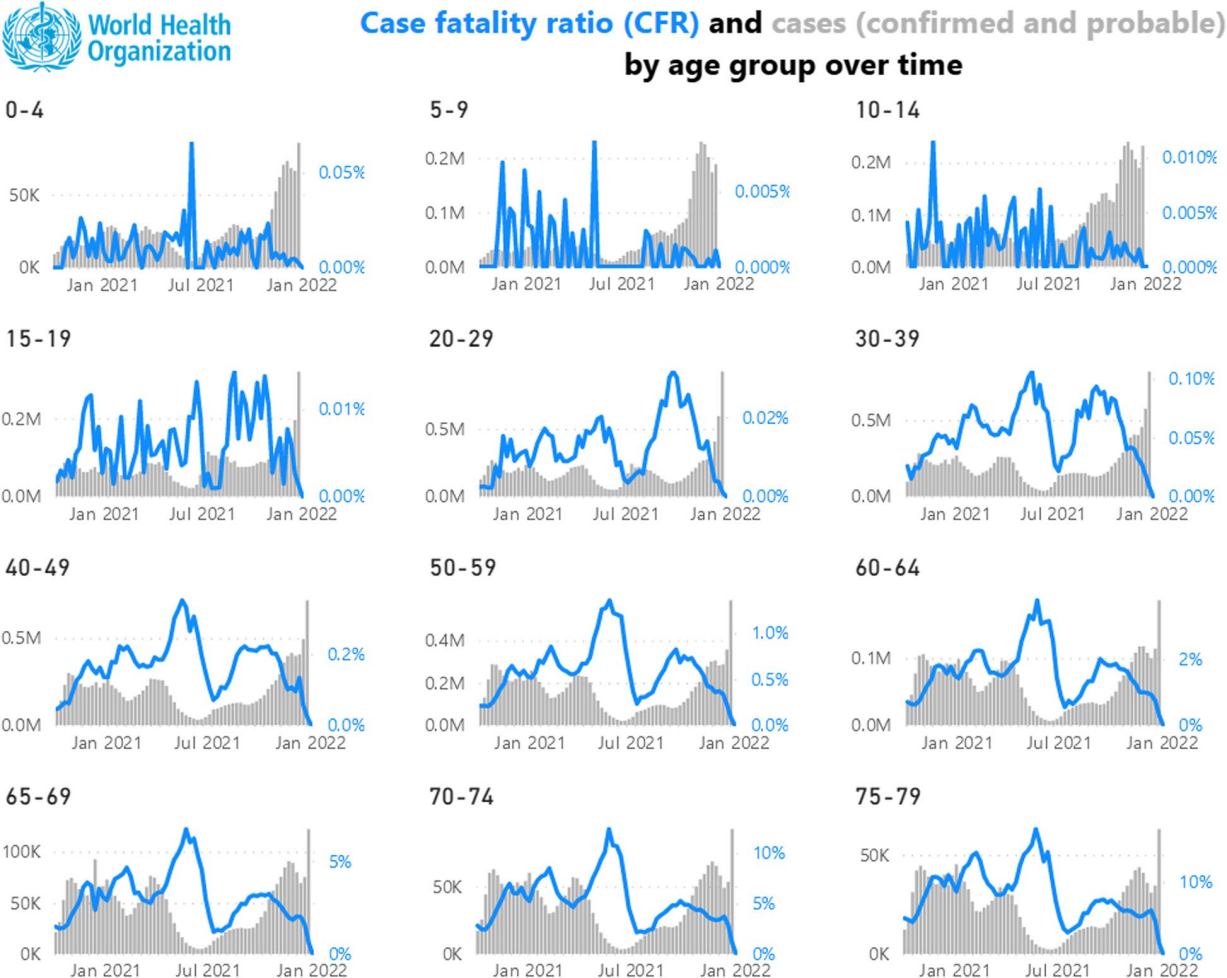
Examples of case fatality ratio and cases trends by age group visualization from the WHO COVID-19 Dashboard

Age disaggregation of case fatality rate allows the detection of unusual mortality trends in specific age groups and the identification of potential high risk groups (Fig. 5).

**Fig. 6:**
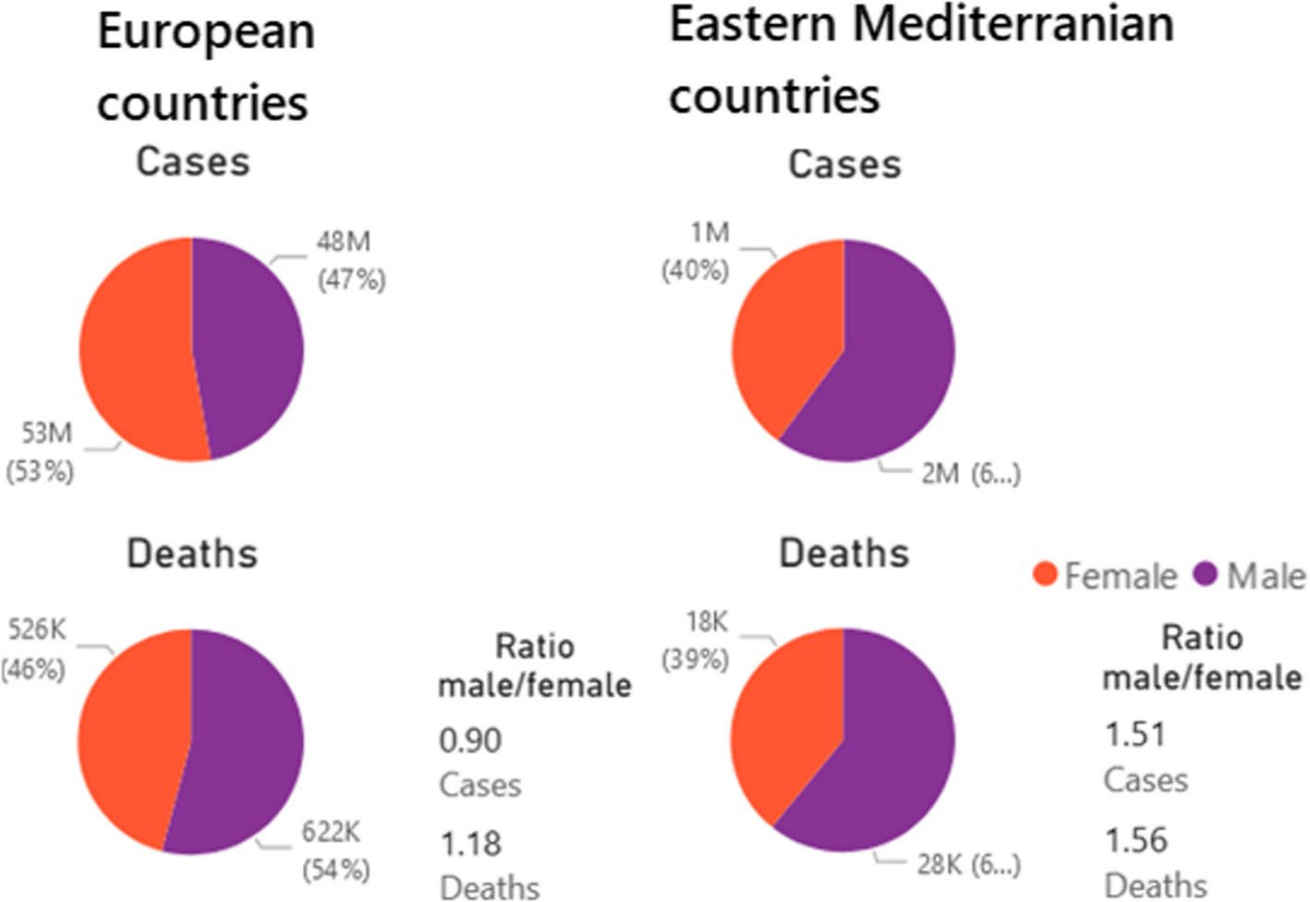
Examples of sex disaggregation of cases and deaths from the WHO COVID-19 Dashboard

Sex disaggregation of cases and deaths (Fig. 6) allows the highlighting of differential risk of exposure and the identification of target groups with higher risk of exposure and severe outcomes, and the subsequential tailoring of Risk Communication and Community Engagement (RCCE), communications, NPI and response. Potential gender-based factors of exposure include social and behavioural factors, and severe disease and death can be due to different physiological and clinical manifestations.

**Fig. 7:**
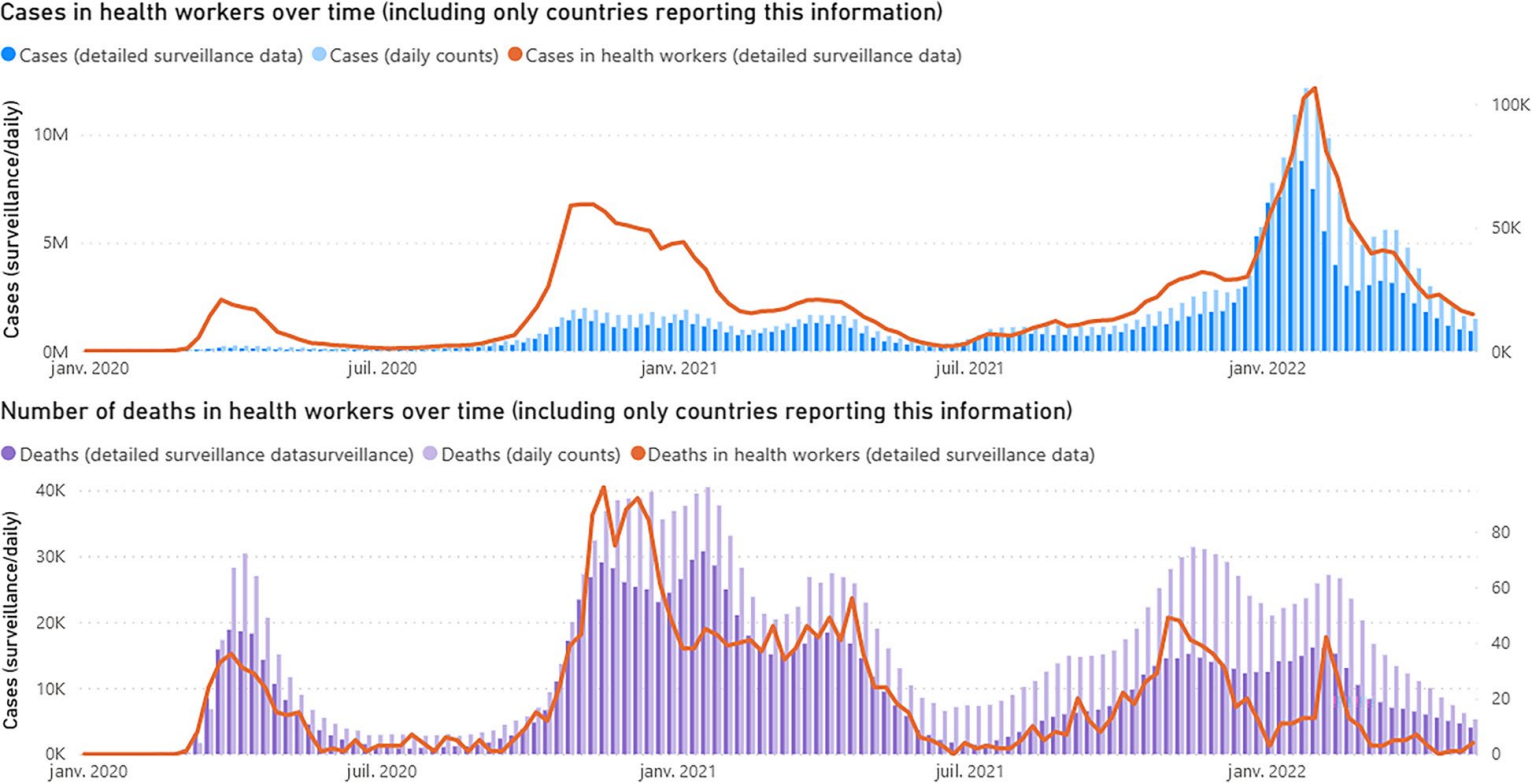
Examples of cases and deaths among Health Care Workers visualization from the WHO COVID-19 Dashboard

Trends in HCW infections regarding trends in general population (Fig. 7) allow to detect differential risks in infection and fatal outcome in HCWs, due to potential increased exposure.

**Fig. 8:**
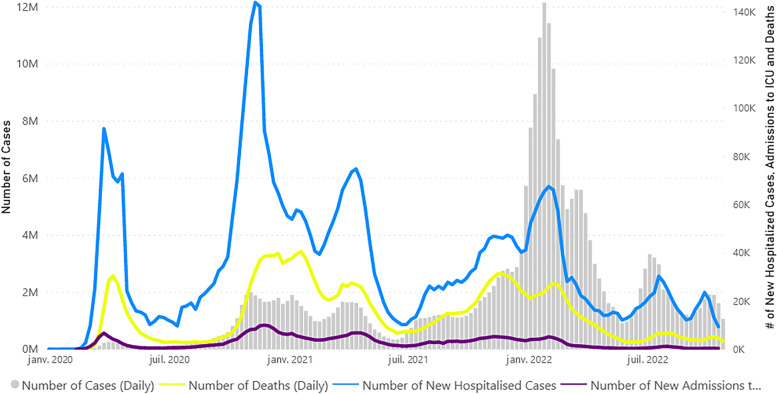
Example of number of new hospitalized cases visualization from the WHO COVID-19 Dashboard

Longitudinal analysis of hospitalization and deaths include trend decoupling between cases, hospitalization, and deaths (Fig. 8), which infers links between severity of clinical manifestation, and burden on healthcare systems.

### Utility and discussion

#### Data platform

It became quickly evident in the beginning of the COVID-19 pandemic that with the volume of cases, a switch from spreadsheet data collection to a more stable system was vital. The WHO has its own warehousing solution with WHO xMART, which allows for data integration, curation, storage, and harmonization of data from different sources and formats to standard models. Access to the data through the web-based WHO xMart user interface was configured in a very granular way from overall data management capacity to only allowing data upload access for selected datasets or permission to only view selected tables. The system was continuously enhanced to resolve issues identified and to fulfill needs for effective COVID-19 surveillance data management on WHO xMart. Thus, the system facilitated collaboration across WHO offices, helped to build and maintain the WHO-HQ COVID-19 surveillance database despite changing in reporting recommendations and to automate data flows. The user interface improved significantly over time, but further efforts to complement data management functions and to ease the development of upload pipelines would be beneficial.

#### Current data availability, coverage, and completeness

Data availability and participation in the system was variable over time and some data only became available retrospectively, but in comparison to the influenza A (H1N1) pandemic in 2009, the collection, aggregation and dissemination of disease surveillance data improved due to higher flexibility of the systems, and collaboration between WHO-HQ, regional offices, and the Global Influenza Programme.

As of 8 March 2022, a total of 184 countries, territories and areas had shared detailed data via case report forms or weekly aggregate surveillance with WHO. Of the 440 million cases reported globally, 327 million cases (74%) were reported with detailed information to WHO-HQ COVID-19 surveillance. Data was reported by sex for over 258 million cases (59%), by age for 268 million cases (61%) and by age and sex combined for 255 million cases (58%). There were also data on COVID-19 cases among health care workers, with just over 4 million cases, and 8700 deaths, recorded in the system.

**Fig. 9:**
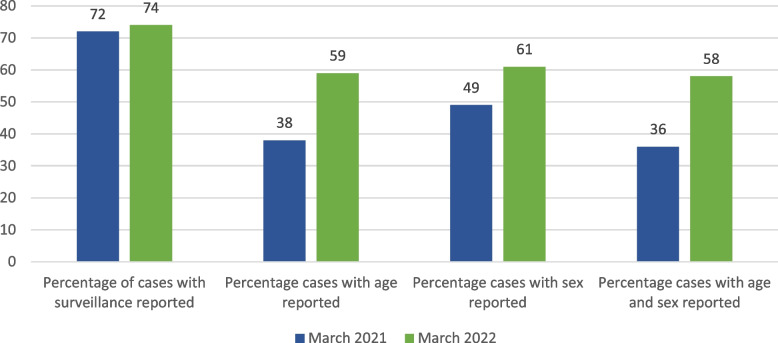
Overall availability of data and data with age and sex disaggregation in the WHO-HQ COVID-19 surveillance database (percentage of countries)

Comparing data from March 2021 (the date of public release of the WHO COVID-19 detailed surveillance dashboard) and March 2022, the proportion of cases for which detailed surveillance data has been reported remained stable, at 72 to 74% respectively, while the overall proportion of cases for which disaggregated data by age and/or sex has been reported increased (see Fig. 9). The completeness of disaggregated data varies considerably across WHO regions and countries, but availability of age and sex disaggregated data has improved overall from 36% of countries in March 2021 to 57% in March 2022.

#### Utility of data disaggregation by age and sex, for health care workers and for hospitalizations

##### Age and sex disaggregation

Age disaggregation enables risk assessment for specific age groups who are considered at risk of severe disease, and death (for instance of infants, children, and people over 65), and can also provide insight on increasing risk of transmission. For example, the data showed that young males in Middle Eastern and Asian countries had higher rates of COVID-19 infection compared to the general population in early 2020. After field investigation, it appeared that these populations were mainly comprised of young male migrant workers, living in crowded conditions [20]. In another example, a greater proportion of young adults as compared to older adults were testing positive for COVID-19 in European countries in the summer of 2021.

Sex disaggregation of cases and deaths showed two patterns: the first was that the majority of cases were in women while the majority of deaths were reported among men; the second was that both cases and deaths were higher among men than women (Fig. 6). Gender-based differences in exposure and access to healthcare and testing facilities have been hypothesised as causes for these differences and are still under investigation. Social and behavioural differences based on gender have also been examined [21–24]. It has been established that men have a higher risk of severe disease and death attributable to COVID-19 than women [25].

Comparative analysis with seroprevalence data allows the comparison of the infections reported by surveillance systems against the real range of infections as detected by seroprevalence. The comparison of the proportion of infections in both methods highlights the proportion of infections that were undetected or unreported by the surveillance system at the time of infection. This method allows the assessment of the ascertainment of COVID-19 surveillance systems, i.e. the capacity of a surveillance system to accurately and timely reflect the reality of disease burden. The proportion of asymptomatic COVID-19 cases who did not seek care was the leading cause of under-ascertainment [26]. These comparisons could potentially highlight under-ascertainment linked to equity factors, for instance in populations with lower access to testing or healthcare.

Going forward, a comparative review of age and sex disaggregated data between epidemiological surveillance and vaccine coverage would allow the investigation of unexpected increases in severe cases and deaths due to suboptimal vaccine coverage in specific groups.

##### Health care workers

Health care workers (HCWs) are a population group with high risk of COVID exposure and have consequently been included as a specific category in surveillance recommendations. The aim of surveillance is to follow trends in HCW infections as compared to trends in the general population and to assess relative risk of infection and death, with the caveat that the place of exposure is not documented for HCWs. According to the WHO COVID-19 surveillance data base, COVID-19 cases among HCWs were higher than the general population in the early stages of the epidemic. However, after Mid 2020, trends in cases among HCWs have been following general population, and the case fatality ratio among HCWs is also lower than among the general population. Several factors explaining these trends are under investigation, including access to testing, access and use of personal protective equipment, a high proportion of women in the health workforce, and the healthy worker effect [27–29].

##### Hospitalizations

The number of new hospitalized cases allows the estimation of the risk of severe disease and hospitalization and anticipation of the burden on healthcare capacity. However, this has shifted with the appearance of the Omicron variant: the large scale of Omicron transmission resulted in an unprecedented number of cases, and while the proportion of severe cases was small, they were still substantial in terms of absolute number of cases and led to hospital capacity being challenged in many parts of the world. Furthermore, it appeared that an increasing volume of hospitalized cases were tested incidentally for COVID-19, and since these were added to the newly hospitalized case tallies the difference in the cause of the hospitalization risked going undetected [30]. At present, hospitalization is a staple indicator in the estimation of burden of disease and is under extreme scrutiny regarding the future of COVID-19 surveillance, in conjunction with healthcare capacity. Notwithstanding this, currently, hospitalization data is still sparse for many countries in the WHO COVID-19 surveillance database.

#### Challenges

##### Surveillance strategy and adaptability to variable changes over time

The scale of the pandemic resulted in changes to surveillance strategies during the pandemic, shifting from case-based data to weekly aggregated data. Furthermore, as Member States and WHO regional offices upgraded and adapted their surveillance strategies, the COVID-19 surveillance data came through WHO regional offices to WHO-HQ in various data flows and data formats, which required assessment for data acquisition automation and occasionally manual data processing to populate the combined dataset. The use of existing influenza systems to collect COVID-19 surveillance data was a great asset, as it contributed to rapid implementation and automation of data flows.

However, the pace of transmission dynamics due to Variants of Concern, such as Omicron, and the scale of the burden of disease hampered the adaptability of surveillance systems to properly assess the impact of vaccine status and seroprevalence on infection and risk of death.

##### Volume of data

The ad hoc data collection solutions implemented at the early stages of the pandemic, such as shared spreadsheets for case-based data, could not accommodate the scale of the pandemic spread and the number of cases at country level, even less at WHO regional and HQ levels given the volume of surveillance data to process and curate. As some countries kept up case-based surveillance, the case-based dataset volume kept increasing. The volume of case report forms warranted a huge scale-up in processing and memory capacity, with the database reaching over 100 million individual case reports. Although the WHO xMart system was continuously adapted and enhanced in order to meet needs, queries to the case-based dataset and data curation of those data are still time-consuming and represent an ongoing challenge and a drawback in data analysis.

##### Sustaining multi-level collaboration with WHO regional offices and member states

The pandemic brought a huge reporting burden on IHR focal points in regional offices and Member States, as well as on public health surveillance systems. WHO-HQ and regional offices have collaborated closely to build the most effective dataflow. This, together with the possibility to tailor the data flows to country needs and practices and keeping a close line with countries, helped greatly to resolve bottlenecks and reporting issues and had a positive impact on the data quality. Standardization data formatting reporting across regional offices has remained a challenge, as some decided to keep the case-based reporting even as weekly aggregated data was encouraged as an attempt to alleviate reporting burden given the scale of the pandemic. Feedback from countries and regions will feed into future updates of global COVID-19 surveillance recommendations. Adaptability at country level to updates in surveillance recommendations, variables, and age groups proved challenging with an estimated six-month delay for countries to roll out new recommendations and report accordingly.

##### Data sensitivity and reporting

While reporting of COVID-19 cases and deaths fell under the IHR legal requirement to be reported to WHO, the reporting of detailed disaggregated data fell under a grey area, resulting in some countries withholding COVID-19 surveillance data. Furthermore, given the impact of epidemiological spread of the disease in Member States on PHSM, international travel and economic response, COVID-19 surveillance data was very sensitive, especially during the emergence phase of Variants of Concern (VOC). Sensitivity on equity issues were noted, such as COVID-19 transmission in children, sex disaggregation of cases and deaths, and hospitalization data. Reporting of surveillance data on HCWs was also particularly sensitive. All those sensitivity issues were directly reflected in data reporting, data completeness and availability of data for analysis.

##### Variability between countries: definitions used for surveillance, completeness of reporting

There is variability between surveillance case definitions applied in countries, as well as testing strategies and eligibility, availability and type of tests varied between countries (RDT versus PCR, etc). Hospitalization data was also reported differently - some countries reported new admissions, some reported current hospitalized cases, others counted only admissions for COVID-19 treatment, while some included incidental COVID-19 cases (tested on admission for medical reasons other than COVID-19). The differences in reporting also vary across time in accordance with transmission dynamics, burden of disease and strain on public health systems. In the WHO COVID-19 surveillance database these inconsistencies are highlighted mainly when comparing the completeness of the detailed surveillance data to the overall number of cases and deaths reported.

On the matter of recording and reporting of deaths related to COVID-19, the WHO definition of COVID-19 death for surveillance purposes [9] is very sensitive and intended to capture broadly deaths associated with COVID for surveillance perspective on the impact of the disease, rather than assess clinical causality, as physiopathology was not well understood in the early stages of the pandemic. Despite this sensitivity, COVID-19 death toll reported to WHO has been widely described as underreported.

Indeed, the underestimation of the death toll has been documented by WHO in the document Revealing the toll of COVID-19 [31], and on the webpage The true death toll of COVID: estimating excess mortality [32]. More recent work on the matter includes COVID’s true death toll: much higher than official records [33] and The pandemic’s true death toll [34].

In accordance with WHO regional offices, the recommended weekly aggregated dataset included metadata on the surveillance strategies and definitions used in the countries, as well as detailed situation reports, but these were seldom reported by the Member States, leading WHO to rely on event-based data and investigation through public health websites to provide context to the data provided and support analysis.

##### Timeliness of data

This database was not intended to provide real-time data but rather provide a macro-level understanding of the trend over time. Thus data is reported with a delay ranging between a week and several months, and has been monitored as a quality attribute that has improved over time.

### Lessons learnt and recommendations for the future

WHO’s role in a PHEIC, including surveillance recommendations and guidance dissemination, as well as the network of WHO offices and Member States confer a unique advantage for data collection and dissemination. The COVID-19 pandemic showed tremendous capacity surge, adaptability and commitment in displaying surveillance data.

Requested by WHO, an external review of WHO COVID-19 surveillance was conducted in 2021 by Resolve to Save Lives and provided a first set of recommendations [35]. Further recommendations on surveillance data acquisition based on the COVID-19 surveillance experience at WHO-HQ include:

- Investment in national disease surveillance strengthening, with technical support from WHO country and regional offices.
- Investment in interoperability of existing systems of different data entry and management tools to further ease integration of data from different settings.

Agreement on a staple minimal outbreak dataset, integrated in existing surveillance systems (such as Early Warning Alert and Response Systems EWARS [36], DHIS2 [37], the Outbreak Toolkit [38]), which can be adaptable through interconnectivity. In the same spirit and objectives as the HL7 initiative designed for patients care and clinical information [39], the outbreak toolkit project developed a list of variables that represent the essential and non-exhaustive information needed to investigate an outbreak. WHO supported by an international working group is proposing a list of essentials variables and its standards. Those variables were included into a standard questionnaire, the T0 questionnaire available on WHO website (https://www.who.int/emergencies/outbreak-toolkit/data-collection-standards/t0-initial-case-investigation-form). This T0 questionnaire was used to develop the initial case reporting form for COVID-19 case based surveillance that WHO shared with regions and countries as soon as 21 of January 2020. Description of the process to identify essential variables is provided in the article: Data collection for outbreak investigations: Process for defining a minimal data set using a Delphi approach [40]. The Outbreak toolkit project aims at providing tools and standards to increase interoperability of databases and facilitate the work of field epidemiologist investigating outbreak in remote settings: https:/www.who.int/emergencies/outbreak-toolkit

- Investment in existing systems and data platforms and enforcement of emergency Standard Operating Procedures, to ensure agility and surge reactivity to implement data collection requirements during an emergency.
- Investment in human resources data management and analytical skills and institutional capacity to ensure surge reactivity.
- Identification and implementation of a strategic information plan including data standards, data sharing, and minimum reportable data by Member States, that builds on existing systems, is scalable and can inform evidence-based decision-making across Member States to develop global public health information goods during major events.
- Age and sex disaggregation, as well as a focus on vulnerable groups, should be included in the minimum reportable dataset to provide equity insight on transmission dynamics and risk factors, especially in in lower income countries and fragile settings.
- Centralization of emergency health information system with shared data management responsibilities designed for global events and potential Public Health Emergencies as to contribute to timely curation and availability of data and to ensure consistency and longitudinal comparability of data analysis.

Setting up a centralized database during a PHEIC may take a few weeks as it requires agreement on the minimum datasets, frequency of reporting, setting up the data pipelines, communication and training of the regions, and data validation. As part of preparedness for future outbreaks with known or unknown pathogens, it would be still important to actively collect data even before such system can be set up to characterize the epidemiological features to inform prompt decision-making.

## Conclusions

Two years after the pandemic was declared, SARS-CoV-2 continues to spread, and is still considered a PHEIC. Ensuring a responsive and relevant global platform for COVID-19 surveillance, although challenging at the global level, has paramount importance for both prospective planning and for retrospective longitudinal studies. To this end, the WHO-HQ COVID-19 surveillance database is continuously maintained, and data analyzed and displayed in the public dashboard. Important aspects of pandemic risk and response have become apparent through analysis of disaggregated data even as reporting completeness, standardization and timeliness of data sharing remain a major constraint for lower income countries and fragile settings. While WHO offices have demonstrated tremendous adaptability and commitment to process COVID-19 surveillance data, lessons learnt from this major event would greatly serve to enhance capacity and preparedness at every level, as well as institutional empowerment towards improvement of shared public health evidence during a PHEIC, with a focus on equity.

## Data Availability

The dataset(s) supporting the conclusions of this article is(are) available in the [repository name] repository : published dashboard [18]: Data in this report are combined from the following sources: - Official reporting to WHO through regional offices: case report forms (CRFs) *(referred to as data source CRF)* [41]. - Official reporting to WHO (HQ and regional offices: daily cases/deaths count *(referred to as data source DAILY or “daily counts”)* [10]. - Official reporting to WHO: weekly aggregate reporting (2 versions, switch of systems in October 2020) *(referred to as data source WEEKLY)* [12]. - Our World In Data *(referred to as data source OWID)* [15]. *- FIND (referred to as data source FIND)* [14]. - Taken from official public websites, not officially reported to WHO *(referred to as data source OTHER).* WHO Member States select the reporting system they prefer to use and data from different reporting systems are reconciled. Individual countries, area and territories may decline to allow country-level disaggregation.

## Abbreviations

API: Application Programming Interface
COVID-19: Coronavirus disease 2019
HCW: Health Care Workers
IHR: International Health Regulations
IMST: Incident Management Support Team
NPI: Non Pharmaceutical Intervention
PHEIC: Public health emergency of international concern
PHSM: Public health and social measures
RCCE: Risk Communication and Community Engagement
SARS-CoV-2: Severe acute respiratory syndrome coronavirus 2
UNICEF: United Nations International Children’s Emergency Fund
WHO: World Health Organization
WHO-HQ: World Health Organization Headquarters
VOC: Variant of concern
