# Correction to: The World Health Organization COVID-19 surveillance database

**DOI:** 10.1186/s12939-023-01870-1

**Published:** 2023-05-17

**Authors:** Maya Allan, Maja Lièvre, Henry Laurenson-Schafer, Stéphane de Barros, Yuka Jinnai, Sophie Andrews, Thomas Stricker, Jesus Perez Formigo, Craig Schultz, Anne Perrocheau, Julia Fitzner

**Affiliations:** WHO-HQ WHE COVID-19 IMST, Geneva, Switzerland

**International Journal for Equity in Health (2022) 21(Suppl 3):167**

10.1186/s12939-022-01767-5

After publication of this article [1], the authors reported that

- The statement in the Funding information section was incorrectly given as ‘The WHO surveillance database and dashboards are funded by WHO funds, including publication charges’ and should have read ‘The WHO surveillance database and dashboards are funded by the WHO. Funding for the journal special issue has been provided by Global Affairs Canada (GAC).‘
- The author name Henry Laurenson-Schafer was incorrectly written as Henry Laurenson-Schaefer.

The original article [1] has been corrected.

## References

[1] Allan M, Lièvre M, Laurenson-Schafer H (2022). The World Health Organization COVID-19 surveillance database. Int J Equity Health.

